# Protective Role of Plant Sterol and Stanol Esters in Liver Inflammation: Insights from Mice and Humans

**DOI:** 10.1371/journal.pone.0110758

**Published:** 2014-10-30

**Authors:** Jogchum Plat, Tim Hendrikx, Veerle Bieghs, Mike L. J. Jeurissen, Sofie M. A. Walenbergh, Patrick J. van Gorp, Els De Smet, Maurice Konings, Anita C. E. Vreugdenhil, Yasmin Dias Guichot, Sander S. Rensen, Wim A. Buurman, Jan Willem M. Greve, Dieter Lütjohann, Ronald P. Mensink, Ronit Shiri-Sverdlov

**Affiliations:** 1 Department of Human Biology, School for Nutrition, Toxicology and Metabolism, Maastricht University, Maastricht, the Netherlands; 2 Department of Molecular Genetics, School for Nutrition, Toxicology and Metabolism, Maastricht University, Maastricht, the Netherlands; 3 Department of Pediatrics, School for Nutrition, Toxicology and Metabolism, Maastricht University, Maastricht, the Netherlands; 4 Department of General Surgery, School for Nutrition, Toxicology and Metabolism, Maastricht University, Maastricht, the Netherlands; 5 Institute of Clinical Chemistry and Clinical Pharmacology, University of Bonn, Bonn, Germany; 6 Atrium Medical Center Parkstad, Heerlen, the Netherlands; Nihon University School of Medicine, Japan

## Abstract

The inflammatory component of non–alcoholic steatohepatitis (NASH) can lead to irreversible liver damage. Therefore there is an urgent need to identify novel interventions to combat hepatic inflammation. In mice, omitting cholesterol from the diet reduced hepatic inflammation. Considering the effects of plant sterol/stanol esters on cholesterol metabolism, we hypothesized that plant sterol/stanol esters reduces hepatic inflammation. Indeed, adding plant sterol/stanol esters to a high-fat-diet reduced hepatic inflammation as indicated by immunohistochemical stainings and gene expression for inflammatory markers. Finally, adding sterol/stanol esters lowered hepatic concentrations of cholesterol precursors lathosterol and desmosterol in mice, which were highly elevated in the HFD group similarly as observed in severely obese patients with NASH. In vitro, in isolated LPS stimulated bone marrow derived macrophages desmosterol activated cholesterol efflux whereas sitostanol reduced inflammation. This highly interesting observation that plant sterol/stanol ester consumption leads to complete inhibition of HFD-induced liver inflammation opens new venues in the treatment and prevention of hepatic inflammation.

## Introduction

NASH is generally recognized as the hepatic event of the metabolic syndrome. The current prevalence of NASH within the general population is estimated to be as high as 2–3%. However, among obese subjects the prevalence is far higher [1], and therefore the number of NASH patients is expected to increase dramatically due to the increasing prevalence of obesity. Most importantly, not only adults are at risk, but also the increasing prevalence of child obesity is a major threat. It is generally accepted that steatosis is probably benign and reversible, whereas the introduction of inflammation can lead to further progression into NASH, ultimately resulting in liver fibrosis, cirrhosis and in some cases eventually liver failure and hepatocellular carcinoma. Pharmacological possibilities to interfere with hepatic inflammation are hardly available and information on dietary determinants is limited. Therefore, there is an urgent need to identify novel (dietary) strategies with the capacity to lower liver inflammation.

Plant sterols and plant stanols are natural dietary ingredients, sharing structural similarities with cholesterol. The average intake of plant sterols from habitual diets is approximately 250 mg/day, which is mainly derived from vegetable oils, grain products, nuts, seeds, fruits and vegetables. The intake of plant stanols (the saturated derivatives) originates from the same sources, but is considerably lower. Quantitatively, the most abundant plant sterols in the human diet are β-sitosterol, campesterol and stigmasterol, while plant stanols are less abundant and consist mainly of sitostanol and campestanol [2]. It is well established that plant sterols and stanols interfere with intestinal cholesterol absorption and the consequent cholesterol-lowering effect of plant sterols was already observed in 1950. Nowadays it is generally accepted that a daily intake of 2.5 g plant sterols or stanols lowers serum cholesterol concentrations up to 10% [3]. While evidence suggests a crucial role of (dietary) cholesterol in hepatic inflammation [4], the role of plant sterol and stanol esters in liver inflammation is not yet established. Considering the beneficial effects of plant sterol and stanol esters on cholesterol metabolism, we hypothesized that consumption of plant sterol and stanol esters will lead to reduced hepatic inflammation. In addition, the recent observation that serum desmosterol concentrations were elevated exclusively in NASH but not in NAFLD patients [5] together with the recent finding of Spann et al [6] suggesting a prominent role for desmosterol as a master regulator of inflammation in macrophages prompted us to test this idea by measuring the same precursor concentrations not only in the livers of the HFD fed mice but also in another cohort of 57 severely obese patients (i.e. patients were classified as control (<5% steatosis), NAFLD or NASH). Using this cohort, we could directly link the data obtained in our hyperlipidemic mouse model for NASH with the human situation making translational assumptions more likely.

In this study, we show for the first time that adding plant sterol or stanol esters to the HFD in hyperlipidemic mice dramatically lowered the development of hepatic inflammation. This protective effect of plant sterol and stanol esters on liver inflammation could open new venues in the treatment or prevention of hepatic inflammation. The fact that we observed a pronounced increase both in hepatic desmosterol and lathosterol concentrations in HFD mice as well as in serum desmosterol and lathosterol concentrations in NASH but not in NAFLD patients, suggested a prominent role for these cholesterol precursors in the pathogenesis of liver inflammation. Interestingly, the increased desmosterol and lathosterol concentrations in the HFD mice were completely absent after plant sterol and stanol ester consumption indicating the preventive nature of these dietary compounds. To further substantiate the direct cellular effects of desmosterol and sitostanol we showed in vitro in LPS triggered BMM that desmosterol activated cholesterol efflux whereas sitostanol reduced inflammation.

## Results

### Plant sterol and plant stanol esters lead to dramatic reduction in hepatic inflammation

**Figure 1:**
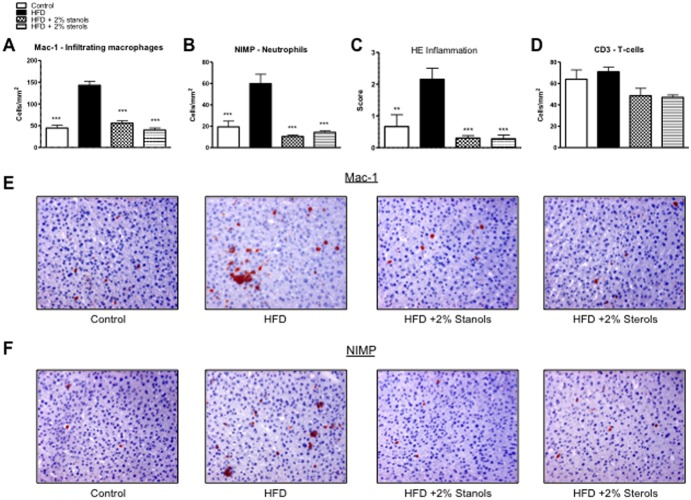
Parameters of hepatic inflammation. (A, B) Liver sections were stained for infiltrating macrophages and neutrophils (Mac-1) and neutrophils (NIMP). From each liver, 6 random pictures were taken at 200x magnification to cover the whole slide. Positive cells for the specific staining were then counted being indicative for inflammation (C) Result of scoring for inflammation by an experienced pathologist using the HE staining in all groups. (D) Liver sections were stained for T-cells (CD3) and positive cells counted. (E, F) Representative pictures of Mac-1 staining and NIMP staining in the four experimental groups (200x magnification). *P<0.05, **P<0.01, and ***P<0.001, respectively.

To investigate the effect of plant sterol and stanol esters on hepatic inflammation, liver sections of mice that consumed the different diets were used for immunohistochemical stainings to detect the presence of infiltrated macrophages (Mac-1), neutrophils (NIMP), and T-lymphocytes (CD3) (Figure 1). In line with the reduced inflammation observed on the Hematoxylin and Eosin (HE) staining (Figure 1C), less infiltrating macrophages (Mac-1) and neutrophils (NIMP) were observed in the livers of mice receiving plant sterol or stanol esters compared to mice receiving only the HFD (Figures 1A+B). T-lymphocyte numbers were not significantly changed upon treatments with plant sterol or stanol esters (Figure 1D).

**Figure 2:**
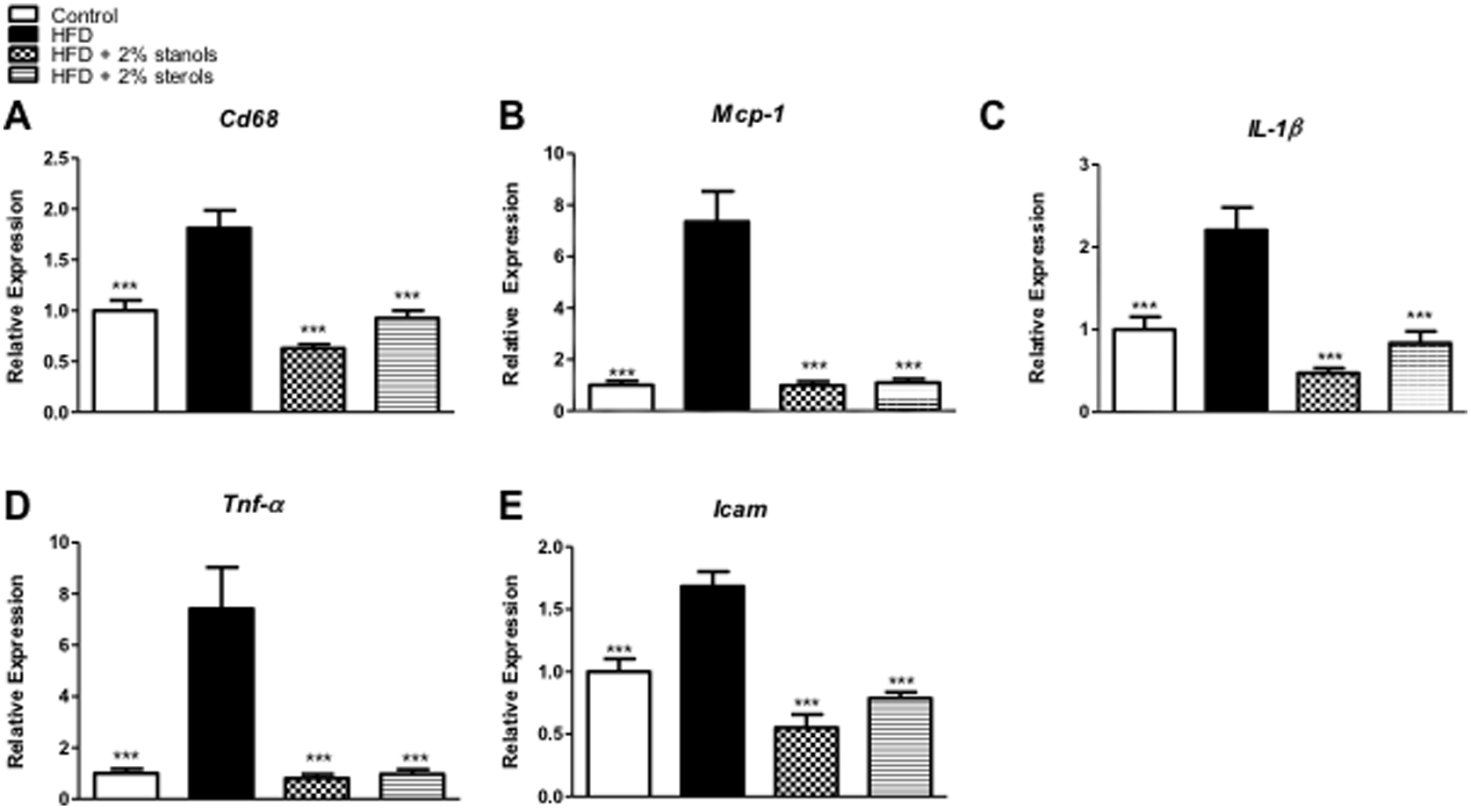
Hepatic gene expression. (A–E) Gene expression analysis of the macrophage marker *Cd68*, monocyte chemoattractant protein 1 (*Mcp-1*), interleukin 1β (*IL-1β*), tumor necrosis factor α (*Tnf-α*) and intercellular adhesion molecule 1 (*Icam*). Relative expression was normalized to endogenous control gene Cyclophilin A. Data were set relative to group on chow diet. n = 10 per group. *P<0.05, **P<0.01, and ***P<0.001, respectively.

To further define the differences in hepatic inflammation, gene expression analysis of the pro-inflammatory markers *Cd68*, *Mcp-1*, *IL-1β*, *Tnf-α* and *Icam* was performed. Importantly, adding plant sterol or stanol esters to the HFD completely blocked the increase in hepatic expression of these inflammatory markers. For each of these inflammatory genes, the expression was significantly lowered in the plant sterol or stanol ester groups compared to the HFD alone and actually returned to values comparable to the chow condition (Figure 2A–E). Altogether, these data indicate the strong inhibitory effect of plant sterol or stanol esters on hepatic inflammation induced by HFD.

**Figure 3:**
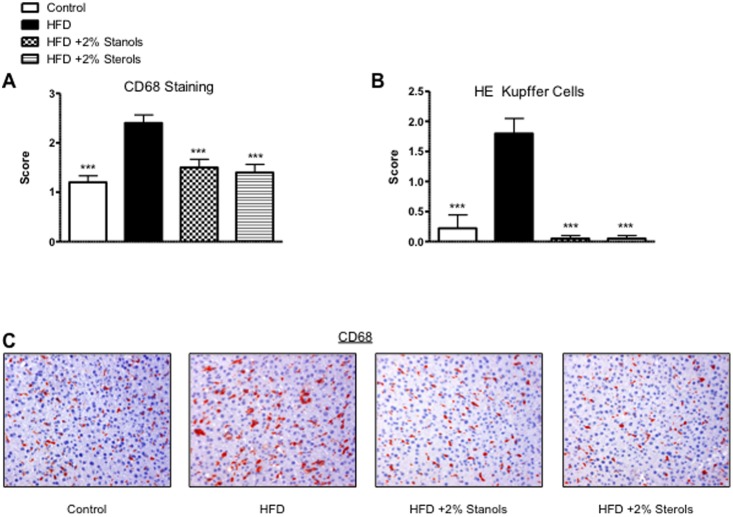
Foamy Kupffer Cells. (A) Scoring for the size and foamy appearance of Kupffer cells using CD68 staining. (B) Scoring for the size and foamy appearance of Kupffer cells using HE staining. A score ranging from 0–3 was given by an experienced pathologist. (C) Representative pictures of the foamy Kupffer cell appearance with CD68 staining (200x magnification). *P<0.05, **P<0.01, and ***P<0.001, respectively.

To determine whether there was a difference in the foamy appearance of Kupffer cells, besides evaluating the *CD68* mRNA expression, we also scored CD68 positive sections, a macrophage marker that stains both Kupffer cells and infiltrated macrophages. In line with the effects described for inflammatory markers, there was a significant increase in the size of CD68 positive cells in the HFD group which was completely reversed to the level of the chow control group after addition of plant sterol or stanol esters to the HFD (Figure 3A–C). Based on this data it is tempting to suggest that the reduced inflammatory response in the livers of mice treated with plant sterol or stanol esters could be ascribed to reduced lipid levels in the Kupffer cells. Altogether, we clearly showed that hepatic inflammation and most likely uptake of lipids into hepatic macrophages was strongly inhibited in the mice receiving plant sterol or stanol ester compared to mice receiving HFD diet alone.

### Plant sterol and plant stanol esters reduce plasma lipid levels, without lowering liver TAG

**Figure 4:**
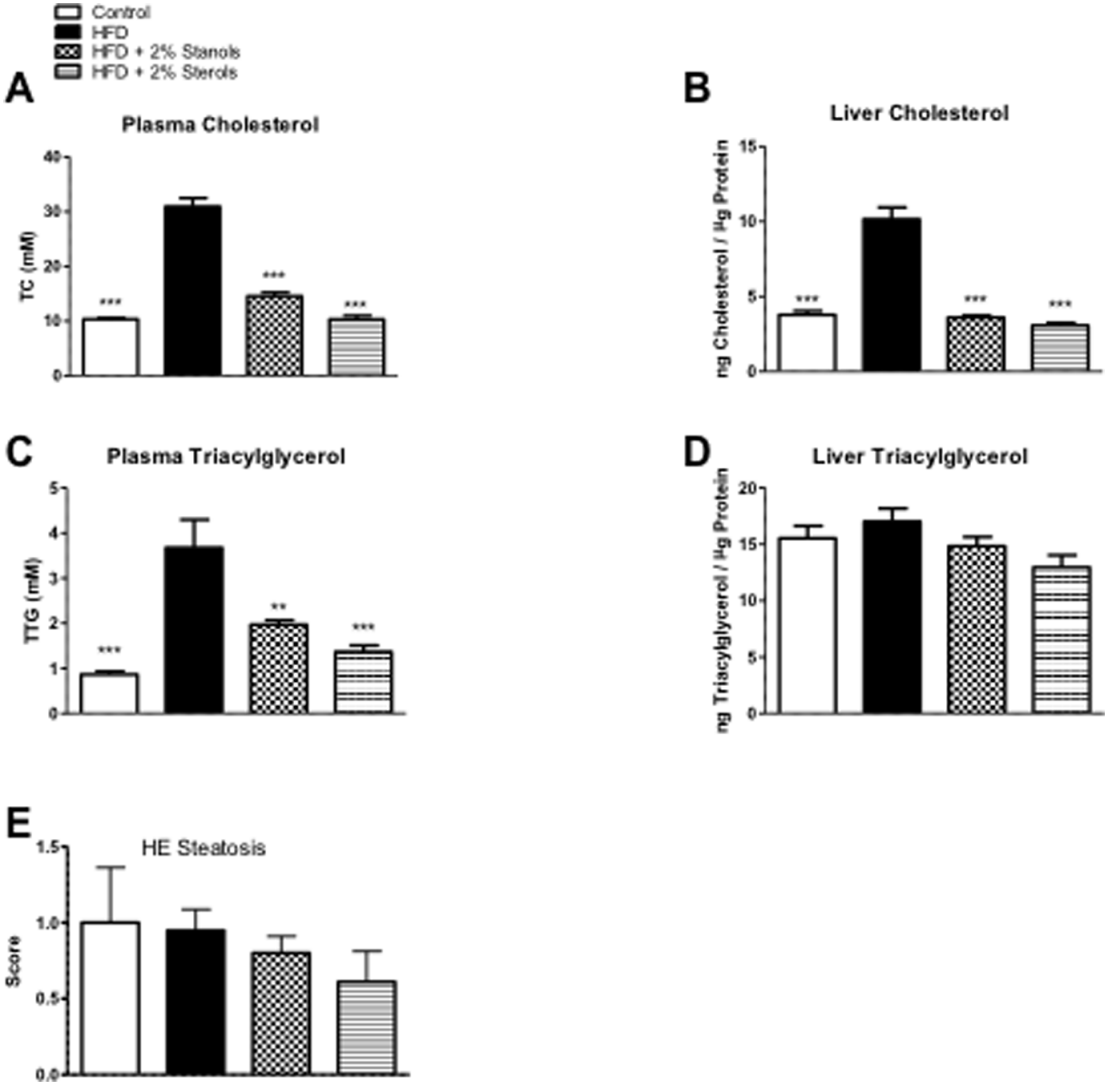
Lipid Measurements. (A, B) Plasma and liver cholesterol measurements. (C, D) Plasma and liver triacylglycerol levels. (E) Scoring of liver slides for the accumulation of fat (steatosis) using HE staining. A score ranging from 0–3 (3 = highest steatosis) was given by an experienced pathologist. *P<0.05, **P<0.01, and ***P<0.001, respectively.

**Figure 5:**
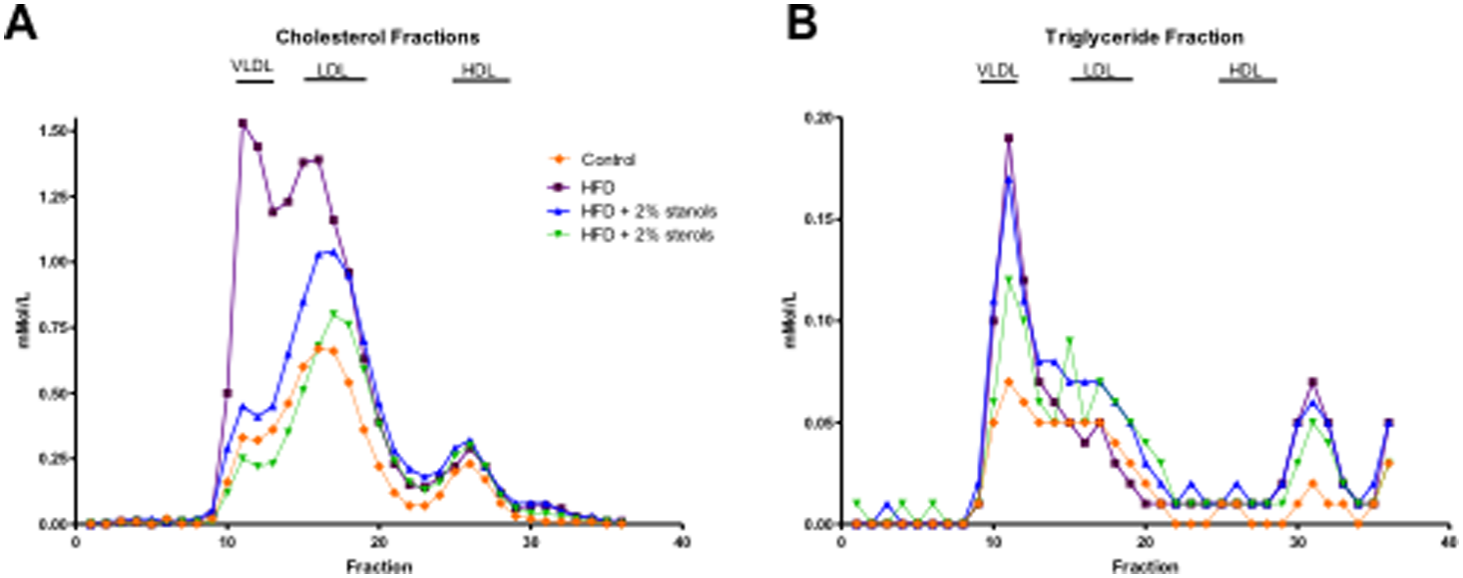
Serum lipid and lipoprotein profiles. (A, B) Using FPLC, serum lipid and lipoprotein profiles were analyzed in all experimental groups. On the chromatogram, the X-axis represents the fractions present in the mixture as a peak, thereby identifying the different components of the mixture. On the Y-axis, the amount of the different fractions can then be read (nMol/l).

As expected, we observed a strong increase in serum and hepatic cholesterol levels of mice fed an HFD as compared to the chow group. Compared to HFD alone, adding plant sterol or stanol esters to the HFD resulted in reduced serum and hepatic cholesterol concentrations to the levels found in controls (Figure 4A+B). As shown in the FPLC profiles (Figure 5A), the reductions in serum cholesterol can be found primarily in the VLDL and LDL fractions. Additionally, we found a reduction in serum TAG concentrations in animals receiving plant sterol and stanol esters enriched HFD (Figure 4C). Surprisingly, despite the effect of plant sterol and stanol esters on plasma TAG concentrations and VLDL particles (Figure 5B), liver TAG concentrations were not significantly different between the groups (Figure 4D).

**Figure 6:**
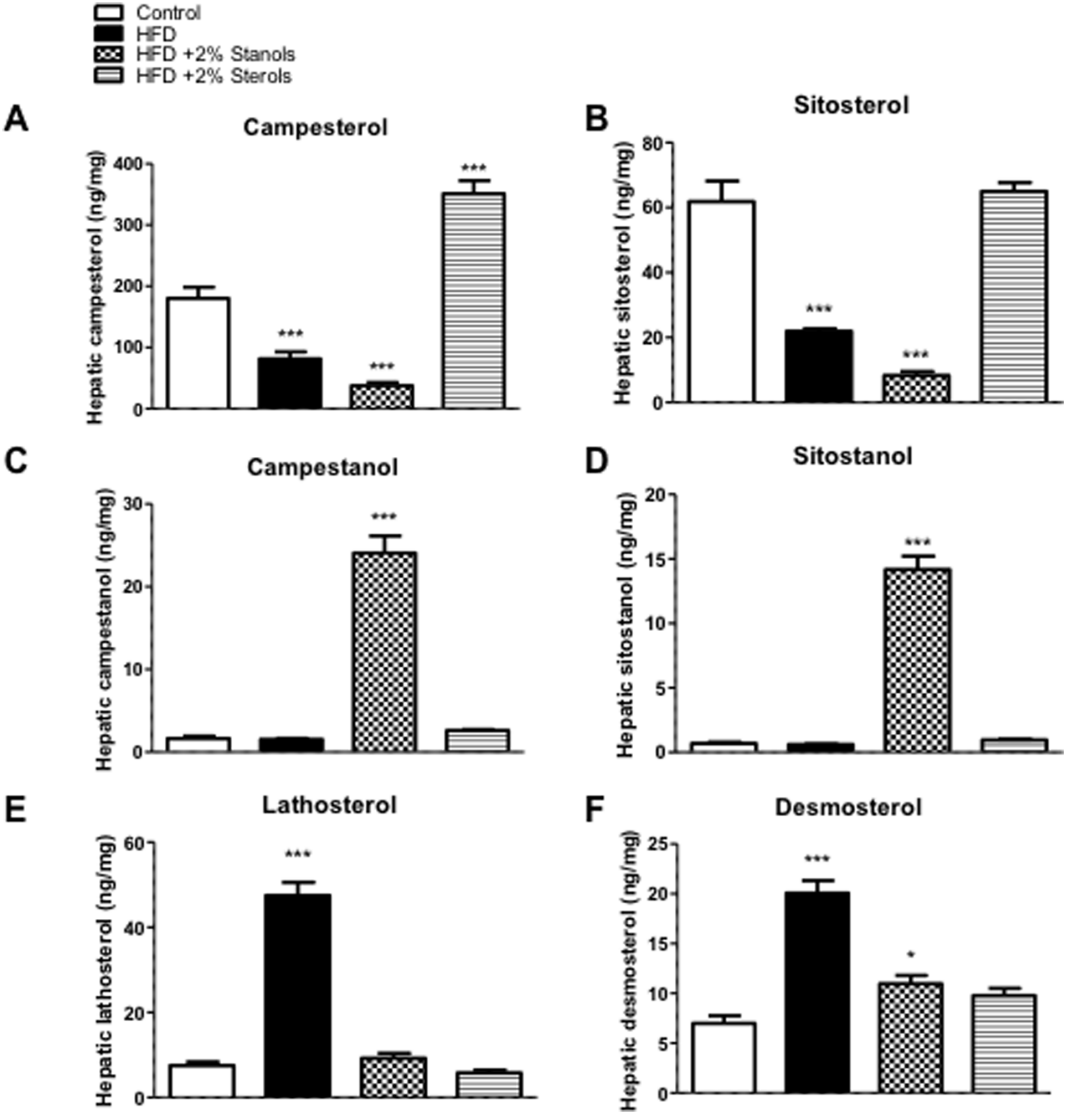
Hepatic non-cholesterol sterol concentrations. Hepatic concentrations of (A) campesterol, (B) sitosterol, (C) campestanol and (D) sitostanol were measured. To analyze endogenous cholesterol synthesis, hepatic (E) lathosterol and (F) desmosterol were measured. All values are shown as absolute concentrations (ng/mg tissue). n = 10 per group. *P<0.05, ***P<0.001.

Upon consumption, it is well accepted that plant sterols and stanols are distributed into different tissues, including the liver [7]. As shown in Figure 6A, hepatic campesterol concentrations increased upon plant sterol feeding and slightly decreased after plant stanol ester feeding. Remarkably, there was no increase in hepatic sitosterol concentrations after plant sterol ester feeding as compared to chow, whereas the expected reduction in hepatic sitosterol concentrations after plant stanol ester feeding was evident (Figure 6B). Regarding hepatic plant stanol concentrations, there was a significant increase in campestanol and sitostanol concentrations after plant stanol ester feeding (Figure 6C, D). This data clearly indicates that hepatic plant sterol and stanol concentrations increase upon consumption and might in theory have local effects. Finally, feeding the HFD severely elevated hepatic lathosterol as well as desmosterol concentrations suggesting a strong increase in endogenous cholesterol synthesis (Figure 6E, F). The recent observations that desmosterol is an important regulator of inflammatory processes in macrophages [6] might also suggest a local effect on inflammation in the Kupffer cells. Moreover, as compared to the HFD, adding plant sterol or stanol esters to the diet lowered hepatic lathosterol and desmosterol concentrations, an indication of a lower endogenous cholesterol synthesis. Finally, hepatic cholestanol concentrations were lowered in the HFD + stanol and sterol ester groups as compared to the HFD group alone (data not shown), indicative of a lowered intestinal cholesterol absorption. Next to that, the expression of enzymes regulating endogenous cholesterol synthesis either via the Kandutsch-Russell pathway or the Bloch pathway suggested increased synthesis in the stanol and sterol ester conditions. As compared to the HFD fed mice, the expression of Cyp51 and LSS was higher both in the sterol and stanol ester condition (Figure S1A, B). Further, the expression of Dhcr24 was increased in the sterol ester condition and the expression of Dhcr7 was increased in the stanol ester condition compared to mice upon HFD (Figure S1C, D). Interestingly, the HFD condition did not show significant differences in expression of these 4 genes compared to chow condition (Figure S1A–D).

### Plant stanols lower Tnf-α secretion *in vitro* in bone marrow macrophages but do not affect expression of lipid transporter genes

**Figure 7:**
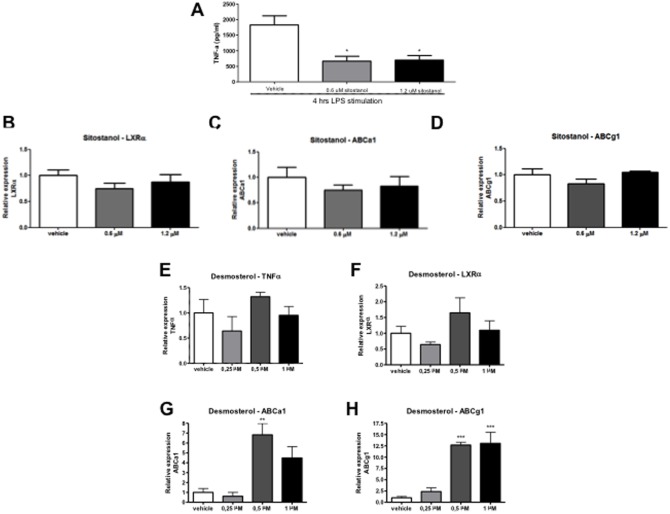
Effect of plant stanols on macrophages *in vitro*. Changes in Tnf-α concentrations in supernatant and LXR target gene expression of bone marrow derived macrophages after incubation with sitostanol (0.6 and 1.2 µm) or desmosterol (0.25, 0.5 and 1.0 µm) and 4 h LPS stimulation. (A) Tnf-α concentrations, (B) *LXRα* mRNA, (C) *Abca1* mRNA, and (D) *Abcg1* mRNA expression after sitostanol exposure, (E) Tnf-α mRNA, (F) *LXRα* mRNA, (G) *Abca1* mRNA, and (H) *Abcg1* mRNA expression after desmosterol exposure. Data were set relative to cells incubated with cyclodextrin (carrier control). *P<0.05; **P<0.01; ***P<0.001.

To investigate whether plant stanols may affect Kupffer cells directly independent of changes in cholesterol or lipid concentrations and the presence of communicating hepatocytes, isolated bone-marrow derived macrophages were incubated with LPS and plant stanols, and levels of Tnf-α secreted in culture medium were measured. Macrophages incubated with plant stanols produced less Tnf-α after exposure to sitostanol, both after 0.6 and 1.2 µm concentrations as compared to cyclodextrin (carrier control) (Figure 7A). On the other hand, there was no significant change in the mRNA expression of liver X receptor alpha (*LXRα*), a transcription factor important for cholesterol homeostasis and lipid transporter genes *Abca1* and *Abcg1* (Figure 7B–D). Since we observed the strong increase in desmosterol concentrations in the livers of the HFD mice, we also cultured the isolated bone-marrow derived macrophages with desmosterol to better understand the effect of these changes. In contrast to sitostanol, supplying desmosterol did not have an effect on the expression of *Tnf-α* (Figure 7E) and cellular Mcp-1 production (data not shown) but instead increased the expression of *LXRα* and the LXR target genes *Abca1* and *Abcg1* (Figure 7F–H) indicating increased cholesterol efflux. Altogether, these data suggest that (1) plant stanols could directly affect inflammation in macrophages independent of lipid and/or cholesterol metabolism and (2) the increased desmosterol concentration in the HFD condition serves to remove excess cellular cholesterol, a situation that was absent in the HDF + plant sterol or stanol ester condition.

### Plasma desmosterol and lathosterol concentrations are elevated in NASH patients

**Figure 8:**
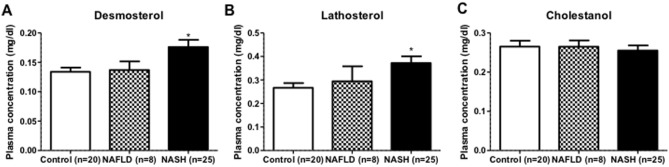
Plasma cholesterol precursor levels in severely obese patients. Serum levels of (A) desmosterol, (B) lathosterol and (C) cholestanol were measured in control (n = 20), NAFLD (n = 8) and NASH (n = 25) patients. All values are shown as absolute levels (mg/dl serum). *P<0.05.

To investigate whether an inflammatory state in the liver is correlated with altered levels of cholesterol precursors in the human setting, plasma desmosterol and lathosterol concentrations were measured in 53 severely obese patients. As shown in Table 1, there were no statistically significant differences in metabolic and clinical parameters between controls, patients with simple steatosis and NASH except from BMI. In Table 2, a detailed description of the histological scoring of the liver biopsies within the NASH group (N = 25) is provided. It appears that we have a mostly mild to moderate NASH population with predominantly steatosis score 2, ballooning score 1, lobular inflammation score 1, and fibrosis score 1. Interestingly, the concentrations of both desmosterol and lathosterol were significantly higher in serum from these NASH patients (n = 25) as compared to patients with simple steatosis (n = 8) or control patients without steatosis (n = 20) (Figure 8A+B). These data are in line with earlier observations in these patients [5], and also aligns with observations in the HFD fed mice and therefore might be suggestive for a link between elevated concentrations of endogenous cholesterol synthesis markers and the presence of liver inflammation. Moreover, serum cholestanol levels were identical between the different patient groups, indicating no difference in fractional cholesterol absorption (Figure 8C).

**Table 1:** Population characteristics.

	Normal	Steatosis	NASH
n	20	8	25
Sex, male/female	8/12	2/6	10/16
Age, y	45±1.7	50±3.2	44±2.1
BMI, kg/m2	42.4±1.5	42.7±2.5	47.9±1.6*
Total cholesterol, mmol/L	4.8±0.2	5.6±0.5	5.2±0.2
HDL, mmol/L	1.1±0.1	1.2±0.1	1.0±0.1
LDL, mmol/L	2.8±0.2	3.1±0.5	3.4±0.2
Triglycerides, mmol/L	1.8±0.3	1.7±0.5	2.0±0.2
Free fatty acids, mmol/L	0.5±0.06	0.6±0.2	0.7±0.06
CRP, mmol/L	9.5±1.8	9.9±3.5	9.5±1.4
ALT, IU/L	23.9±3.5	23.7±4.4	35.3±6.6
AST, IU/L	18.7±2.3	21.8±1.6	28.8±3.7
AST/ALT ratio	0.9±0.1	1.2±0.4	1.0±0.1

Data are represented as mean ± SEM.

*Significantly different from Normal (p<0.05).

**Table 2:** Histological scoring of liver biopsies from NASH subjects.

Brunt score	Definition	NASH subjects(n = 25)
Grade 1	Mild	12
Grade 2	Moderate	11
Grade 3	Severe	2
**Kleiner score**		
Steatosis	<5% (score 0)	0
	5–33% (score 1)	4
	33–66% (score 2)	15
	>66% (score 3)	3
Ballooning	None (score 0)	5
	Few balloon cells (score 1)	20
	Prominent ballooning (score 2)	0
Lobular inflammation	None (score 0)	2
	<2 foci per 200x field (score 1)	14
	2–4 foci per 200x field (score 2)	5
	>4 foci per 200x field (score 3)	4
Fibrosis	None (score 0)/Nondefined	11/3
	Perisinusoidal or periportal (score 1)	6
	Perisinusoidal andportal/periportal (score 2)	3
	Bridging fibrosis (score 3)	2
	Extensive bridging fibrosis, cirrhosis (score 4)	1

## Discussion

Plant sterol- and stanol esters are known for several decades as serum cholesterol-lowering functional food ingredients. However, their effects on hepatic inflammation have never been evaluated. Here we show for the first time the strong ability of dietary plant sterol and stanol esters to suppress the development of hepatic inflammation. The fact that plant sterol and plant stanol esters are natural constituents of food, combined with the lack of adverse side effects upon increased intake in clinical intervention studies, strongly warrants human studies to examine the potential of plant sterol and plant stanol esters as a novel tool for prevention and intervention in of hepatic inflammation. Especially the observation that both in our HFD fed mice as well as in the NASH - but not in simple steatosis - patients desmosterol concentrations are increased, illustrates the validity of our mouse model suggesting the likelihood of extrapolating a successful outcome of such an intervention towards the clinical setting. Moreover, if the increase in hepatic desmosterol concentrationsis is a characteristic of an inflamed liver, the finding that plant sterol or stanol ester consumption showed lower desmosterol and lathosterol concentrations (comparable to the chow condition) is indicative for the protective nature of these dietary compounds.

Considering the beneficial effects of plant sterol and stanol ester consumption on health only from the perspective of serum LDL-cholesterol lowering is without doubt a strong simplification. Interestingly, there are several reports describing effects of plant sterols and stanols on activity of our immune system. For example, our group recently showed that plant stanols induce a Th1 shift in human peripheral mononuclear blood cells from asthma patients, which is most likely due to an activation of the regulatory T-cells [8], [9]. The relevance of this observation in the context of the present study concerns the fact that regulatory T-cells also play a critical role in regulating inflammatory processes in liver inflammation, at least in the situation of NASH.

**Figure 9:**
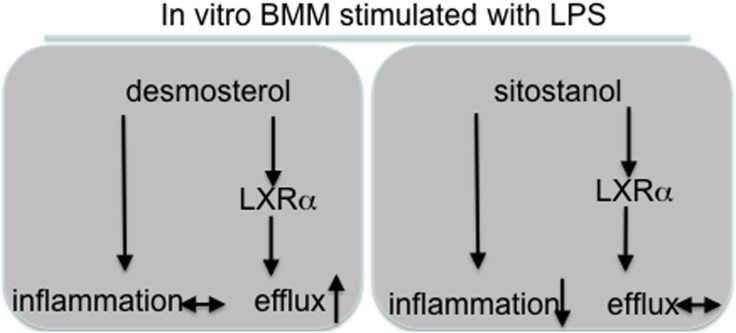
Schematic representation of direct effects of sitostanol vs desmosterol.

Another explanation for the effects of plant sterols and stanols on liver inflammation might relate to the very recent observation that serum desmosterol concentrations were elevated exclusively in NASH but not in NAFLD patients [5]. Indeed we were able to confirm this observation in a cohort of 57 severely obese patients classified either as control (<5% steatosis), NAFLD or NASH. This increase in desmosterol concentrations was also present in our HFD mouse group as compared to the chow fed mice, illustrating that our model mimics human pathology also in this respect. Therefore, since plant sterol or stanol esters in the diet prevent the development of NASH, there is also no build-up of elevated concentrations of cholesterol precursors. The generally accepted assumption is that lowering intestinal cholesterol absorption induces a compensatory increase in endogenous cholesterol synthesis, which is reflected by increased cholesterol precursor concentrations such as desmosterol and lathosterol. This might be different in the condition of inflammation, as our data suggest. Relevantly, Spann et al [6] recently suggested a prominent role for desmosterol as a master regulator of inflammation and cholesterol metabolism in macrophages. In our in vitro experiments in isolated bone-marrow derived macrophages we were indeed able to confirm the effect of desmosterol on cholesterol efflux but not on inflammation. In contrast, sitostanol showed completely the opposite pattern as compared to desmosterol (Figure 9), i.e a reduced inflammatory response but no effect on LXR target gene expression and efflux.

An additional potential explanation to support possible direct effects of plant sterols and stanols on inflammation is related to the finding that plant sterols and stanols can activate LXR. Pharmacological LXR activation was shown to lower dietary cholesterol uptake and to reverse hepatic inflammation [10]. Indeed, both sitosterol and sitostanol were shown to be potent LXR ligands, at least in a cell free *in vitro* system [11], a finding that was later confirmed for other plant sterols like stigmasterol [12]. However, it must be considered that plant sterols and stanols are poorly absorbed from the intestinal tract and the question is whether the increase in hepatic plant sterol or stanol concentrations exceeds the threshold needed to trigger local hepatic LXR activation in the in vivo situation.

A final potential explanation for a direct effect of plant sterols or stanols on liver inflammation relates to their effect on the composition of our intestinal microbiota. Indeed an association between the pathogenesis of NASH and gut microbiota composition has been suggested [13]. Patients with NASH showed a lower abundance of Bacteroidetes as compared to those with only steatosis or healthy controls. Recently, it was described that plant sterol ester intake induced dramatic shifts in the fecal microbiota composition of hamsters showing reductions in Coriobacteriacea and Erysipelotrichaceae [14]. Unfortunately, to the best of our knowledge, these effects have never been studied in humans. Altogether, these data support the notion that plant sterols and stanols affect several different (patho)-physiological processes. We here add another effect suggesting strong anti-inflammatory actions in Kupffer cells. The ultimate challenge is now to see whether these intriguing observations can be extrapolated to the human situation.

Besides the above-mentioned possible direct effects the most likely explanation for the protective effects of plant sterol and stanol ester consumption on hepatic inflammation relates to a changed cholesterol flux from the intestine to the liver. This is fully in line with earlier observations suggesting a crucial role of (dietary) cholesterol in hepatic inflammation [4]. Moreover, Yoneda and coworkers earlier showed in a small pilot experiment that six months ezetimibe treatment (10 mg/day), which lowered intestinal cholesterol absorption pharmacologically, not only improved serum aspartate aminotransferase, alanine aminotransferase, gamma-glutamyl transpeptidase, and high-sensitivity C-reactive protein but most importantly also improvements in histological observations in follow-up liver biopsies in NAS score and steatosis grade [15]. This clearly suggests that cholesterol fluxes are important in this respect.

Interestingly, the observed improvement in hepatic inflammation in the plant sterol and stanol occurred without a change in liver TAG concentrations. These data support our previous observations indicating that progression and regression of steatosis are not correlated with inflammation [4]. In contrast to the unaffected hepatic TAG concentrations there was a highly significant reduction in liver cholesterol concentrations. These data are in line with our previous observations indicating that the inflammatory response in the liver is mainly triggered by the accumulation of cholesterol specifically in the Kupffer cells. Reduced uptake of cholesterol from the diet by Kupffer cells resulted in decreased hepatic inflammation. While inflammation was reduced, the levels of triglycerides in the liver were unchanged indicating equal levels of steatosis. This observation was confirmed in different studies we performed; indicating that not the total levels of triglycerides and cholesterol, but the intracellular distribution of cholesterol is the main trigger for hepatic inflammation [4]. In this context, it was recently suggested that especially the Kupffer cells take up modified cholesterol-rich lipoproteins via scavenger receptors, and due to the accumulation of cholesterol instead of TAG, they become activated and initiate an inflammatory reaction [7], [16]. In line with these assumptions, we also observed a significant reduction in the prevalence of foamy Kupffer cells after adding plant sterol or stanol esters to the HFD.

In conclusion, we here demonstrate that consumption of plant sterol or stanol esters leads to a complete absence of HFD-induced liver inflammation. The fact that hepatic desmosterol concentrations were increased in morbidly obese NASH patients as well as in the HFD mice with liver inflammation suggests that hepatic desmosterol is an indication of hepatitis. Our finding that hepatic desmosterol concentrations are not at all increased in the plant sterol and stanol ester groups is in line with the protective phenotype observed in these groups. This highly significant effect is of great interest since plant sterol or stanol esters may be used as dietary intervention to treat and/or prevent hepatic inflammation.

## Materials and Methods

### Mice

Forty 10–12 weeks old female low-density lipoprotein (LDL) receptor deficient mice (LDLr^−/−^) were housed together in groups of 3 or 4 under standard conditions having ad libitum access to food and water. Ten mice were consuming plant sterol poor chow diets, whereas the remaining 30 mice received a plant sterol poor high fat diet (HFD) containing beef fat for 3 weeks. The composition of the three experimental high fat and chow diets is presented in Table 3. After these 3 weeks these 30 mice were randomly allocated to one of the 3 experimental groups (n = 10). The first group continued using the HFD for another 3 weeks, while the second and third groups used the same HFD but now enriched with plant sterol esters (2%) or plant stanol esters (2%). The chow group continued the same plant sterol poor chow diet and served as control. Experiments were performed in accordance with Dutch law for animal experimentation and approved by the Committee for Animal Welfare of the University of Maastricht. Collection of blood and tissue specimens, biochemical determination of plasma and liver lipids, RNA isolation, cDNA synthesis and qPCR were performed as described previously [7], [17]. Hepatic plant sterol and stanol as well as cholesterol precursor concentrations were quantified by GC-MS as described (18).

**Table 3:** Composition of the experimental and chow diets.

	HFD^1^	HFD + Plant sterol esters	HFD + Plant stanol esters	Chow
	Composition (%)			
Sucrose	39.75	38.97	38.97	29.38
Casein	23.64	23.18	23.18	20.00
Beef fat	15.78	15.47	15.47	-
Cellulose	5.91	5.79	5.79	5.00
Olive Oil^4^	2.94	2.07	2.07	2.00
Soybean oil^4^	2.27	2.07	2.07	2.00
Corn Starch	2.59	2.54	2.54	35.92
Vitamin Mix^2^	0.58	0.58	0.58	0.50
Mineral Mix^3^	5.44	5.33	5.33	4.60
Choline	0.47	0.46	0.46	0.40
DL Methionine	0.24	0.23	0.23	0.20
Cholesterol^4^	0.20	0.20	0.20	-
Linseed Oil^5^	0.19	-	-	-
Plant sterol esters^6^	-	3.10	-	-
Plant stanol esters^6^	-	-	3.10	-

1 HFD: high fat diet;

2 Vitamin mix: vitamins premix, trace elements premix;

3 Mineral mix: calcium hydrogen phosphate, calcium carbonate, potassium chloride, potassium dihydrogen phosphate, magnesium sulphate heptahydrate, sodium chloride, magnesium oxide;

4 This added amount of 0.2% cholesterol together with the 0.015% cholesterol from beef fat makes that the diet contains 0.22% cholesterol;

5 The small amounts of olive oil, soybean oil and linseed oil were added to the HFD and not to the HFD + sterol or stanol esters to make the amount and type of fatty acids in the three HF diets comparable since the fatty acids in the sterol and stanol esters (rapeseed oil fatty acids) become available during digestion.

6 The 3.1% plant sterol or stanol esters correspond to ±2% free plant sterols or stanols. The plant stanols used are a mixture of mainly sitostanol and campestanol 85/15 and the plant sterols used are a mixture of mainly sitosterol and campesterol 70/30.

The chow diet contains ±10.2 en% fat, whereas the HFD contains ±41.5 en% fat.

### Lipid analysis

Approximately 50 mg of frozen liver tissue was homogenized as described previously [7], [17]. Both plasma and liver lipid levels were measured with enzymatic color tests (1489232, cholesterol CHOD-PAP, Roche, Basel,Switzerland; TR0100, TG GPO-trinder, Sigma Aldrich, Sigma Aldrich, St. Louis, MO, USA; 999-75406, NEFAC, ACS-ACOD, Wako Chemicals, Neuss, Germany) as was described before [7], [17].

### Liver histology

Frozen liver sections (7 µm) were fixed in acetone and stained with the macrophage and neutrophil marker Mac1 (M1/70), neutrophil marker NIMP, T-cell marker CD3, Kupffer cell marker CD68 (FA11). Paraffin embedded liver sections (4 µm) were stained with Hematoxylin-Eosin (HE). Pictures were taken with a Nikon digital camera DMX1200 and ACT-1 v2.63 software from Nikon Corporation. Immune cells were counted in six 200x microscopical views and were noted as cells/mm^2^.

### GC-MS

Plant sterol (sitosterol, campesterol), plant stanol (sitostanol, campestanol), cholesterol precursor (lathosterol and desmosterol), and cholestanol concentrations were analyzed by gas–liquid chromatography–mass spectroscopy (GC–MS) as described previously [18].

### Quantitative PCR

Quantification of gene-expression of inflammation markers was done by quantitative PCR on a Bio-Rad MyIQ with the IQ5 v2 software (Bio-Rad, Hercules, CA, USA) by using IQ SYBR Green Supermix with fluorescein (170-5006CUST, Bio-Rad, Hercules, CA, USA) and 10 ng of cDNA. For each gene a standard curve was generated with a serial dilution of a liver cDNA pool. To standardize for the amount of cDNA, Cyclophillin A (Cyclo) was used as reference gene. Primer sets for the selected genes were developed with Primer Express version 1.5 (Applied Biosystems) using default settings. Primer sequences:

MCP1-forward, 5′ - GCTGGAGAGCTACAAGAGGATCA - 3′;
MCP1-reverse, 5′ - ACAGACCTCTCTCTTGAGCTTGGT - 3′;
CD68-forward, 5′ - TGACCTGCTCTCTCTAAGGCTACA - 3′;
CD68-reverse, 5′- TCACGGTTGCAAGAGAAACATG - 3′;
TNFa-forward, 5′ - CATCTTCTCAAAATTCGAGTGACAA - 3′;
TNFa-reverse, 5′ - TGGGAGTAGACAAGGTACAACCC - 3′;
Cyclo-forward, 5′ - TTCCTCCTTTCACAGAATTATTCCA - 3′;
Cyclo-reverse, 5′ - CCGCCAGTGCCATTATGG - 3′.
IL1β-forward, 5′ - AAAGAATCTATACCTGTCCTGTGTAATGAAA - 3′;
IL1β-reverse, 5′ - GGTATTGCTTGGGATCCACACT - 3′;
ICAM-forward, 5′ - CTACCATCACCGTGTATTCGTTTC - 3′;
ICAM-reverse, 5′- CGGTGCTCCACCATCCA - 3′;
LXRα-forward, 5′ - CAACAGTGTAACAGGCGCT - 3′;
LXRα-reverse, 5′ - TGCAATGGGCCAAGGC - 3′;
ABCa-forward, 5′ - GCGAGGGCTCATCGACAT - 3′;
ABCa-reverse, 5′ - GAAGCGGTTCTCCCCAAAC - 3′;
ABCg-forward, 5′ - TCGGACGCTGTGCGTTTT - 3′;
ABCg-reverse, 5′- CCCACAAATGTCGCAACCT - 3′;
Cyp51-forward, 5′ - CCACGCTGCCTGGCTATT - 3′;
Cyp51-reverse, 5′ - CTATCCCTGCGCCTGAAACT - 3′;
Lss-forward, 5′ - GCGGCTGTGCGATGCT - 3′;
Lss-reverse, 5′ - AGTAACCCCCACGCTTCTTCTC - 3′;
Dhcr24-forward, 5′ - CACAGGCATCGAGTCATCGT - 3′;
Dhcr24-reverse, 5′- GGCACGGCATAGAACAGGTC - 3′;
Dhcr7-forward, 5′ - CCAAAGTCAAGAGTCCCAACGG - 3′;
Dhcr7-reverse, 5′ - ACCAGAGGATGTGGGTAATGAGC - 3′;
Data from qPCR was analyzed according to the relative standard curve method.

### In vitro studies in bone marrow derived macrophages

To evaluate whether effects of plant sterols and stanols on inflammatory parameters in the liver were present without the potentially interfering effect of changes in cholesterol and lipid concentrations, bone marrow derived macrophages were cultured with and without sitostanol as a typical example of plant sterols or stanols. For this, bone marrow derived macrophages were isolated from the tibiae and femurs of C57BL/6 mice. Cells were cultured in RPMI-1640 (GIBCO Invitrogen, Breda, the Netherlands) with 10% heat-inactivated fetal calf serum (Bodinco B.V. Alkmaar, the Netherlands), penicillin (100 U/ml), streptomycin (100 µg/ml) and L-glutamine 2 mM (all GIBCO Invitrogen, Breda, the Netherlands) supplemented with 20% L929-conditioned medium (LCM) for 8–9 days to generate bone marrow-derived macrophages, as described previously [19]. After attachment, the macrophages were seeded at 350000 cells per well in 24 wells plates and incubated 24 hrs with medium (control), cyclodextrin (carrier control), 0.6 µM sitostanol or 1.2 µM sitostanol. Then cells were washed and stimulated with LPS (100 ng/ml) for 4 hours. Finally, the supernatant was frozen until cytokine analysis and the cells were lysed for mRNA expression analysis. In additional experiments using the same set up, cells were cultured with desmosterol 0.25, 0.5 and 1.0 µM.

### Patient population

Fifty-three severely obese patients undergoing bariatric surgery at the Maastricht University Medical Centre were included. The study was approved by the local Ethics Committee of Maastricht University and conducted in line with the 1975 Declaration of Helsinki guidelines and the Seoul 2008 amendments. All subjects gave written informed consent. Plasma samples and liver wedge biopsies were obtained as previously described [20]. Biopsies were evaluated for histological features criteria of Brunt [21] and Kleiner [22] by an experienced pathologist.

### Statistics

Results are presented as mean ± standard error of the mean (± SEM). Differences between groups were assessed by ANOVA and significant effects were analyzed by post hoc Bonferroni corrections. All analyses were performed using a commercially available statistics package (GraphPad Prism version 5; GraphPad Software Inc, San Diego, CA, U.S; www.graphpad.com).

## Supporting Information

Figure S1 **Hepatic gene expression.** (A–D) Gene expression analysis of enzymes involved in cholesterol biosynthetic pathway *Cyp51*, *LSS*, *Dhcr24* and *Dhcr7*. Relative expression was normalized to endogenous control gene Cyclophilin A. Data were set relative to group on chow diet. n = 10 per group. *P<0.05 and **P<0.01, respectively.
(TIFF)
